# Integrating accelerometry, GPS, GIS and molecular data to investigate mechanistic pathways of the urban environmental exposome and cognitive outcomes in older adults: a longitudinal study protocol

**DOI:** 10.1136/bmjopen-2024-085318

**Published:** 2024-12-10

**Authors:** Ruth F Hunter, Claire Cleland, Mike Trott, Sean O’Neill, Hüseyin Küçükali, Shay Mullineaux, Frank Kee, Jennifer M McKinley, Charlotte Neville, Leeanne O'Hara, Calum Marr, Michael McAlinden, Geraint Ellis, Amy McKnight, Jasper Schipperijn, Joanna McHugh Power, Trung Duong, Bernadette McGuinness

**Affiliations:** 1 Centre for Public Health, Queen's University Belfast, Belfast, UK; 2 School of Natural and Built Environment, Queen's University Belfast, Belfast, UK; 3 Department of Sports Science and Clinical Biomechanics, University of Southern Denmark, Odense, Denmark; 4 Department of Psychology, Maynooth University, Maynooth, Ireland; 5 Institute of Electronics, Communication and Information Technology, Queen's University Belfast, Belfast, UK

**Keywords:** Aging, Dementia, EPIDEMIOLOGY, GENETICS, PUBLIC HEALTH

## Abstract

**Abstract:**

**Introduction:**

Maintaining cognitive health in later life is a global priority. Encouraging individuals to make health behaviour changes, such as regular physical activity, and providing supportive urban environments can help maintain cognitive health, thereby preventing or delaying the progress of dementia and cognitive decline. However, the mechanistic pathways by which the urban environmental exposome influences cognitive health outcomes are poorly understood. The aim of this study is to use granular measures of the urban environment exposome (encompassing the built, natural and social environment) and physical activity to explore how these interact with a person’s biology to ultimately influence cognitive health outcomes.

**Methods and analysis:**

This ongoing study uses a cohort design, recruiting participants from the Northern Ireland Cohort for the Longitudinal study of Ageing and the Harmonised Cognitive Assessment Protocol study. Participants (n=400 at each wave) will be aged ≥65 years and have the capacity to provide written informed consent. Measures include device-measured physical activity (Actigraph wGT3XP-BT), environmental location data (Global Positioning System, Qstarz BT-Q1000XT), linked to a battery of neuropsychological tests, including the Mini Mental State Examination and the Centre for Epidemiological Studies Depression Scale. Blood-derived biochemical, genetic and epigenetic data will be included in multimodal analyses. These data will be integrated with urban environment Geographic Information System data and analysed using causal inference and mediation methods to investigate plausible mechanistic pathways.

**Ethics and dissemination:**

This study has been approved by the Queen’s University Belfast, Faculty of Medicine, Health and Life Sciences Research Ethics Committee (MHLS 21_72). Alongside peer-reviewed publications in high-ranking international journals, dissemination activities include conference presentations, project videos, working papers, policy briefing papers, newsletters, summaries and case study stories.

**STRENGTHS AND LIMITATIONS OF THIS STUDY**

- A strength is the use of device-measured physical activity and the integration of Geographic Information System and Global Positioning System data, combined with cognitive health data.
- The large sample enables an in-depth analysis of the causal mechanisms of cognitive decline, mild cognitive impairment and dementia in older adults.
- The environmental exposure data facilitate the analysis of the impact of co-exposures on cognitive health outcomes and their mechanistic pathways.
- A limitation is the generalisability of results, due to participants residing in Northern Ireland, and the age restriction of over 65 years.

## Introduction

 Maintaining cognitive health and preventing dementia is a global health priority due to the rapidly ageing nature of the population and the increased number of individuals living with mild cognitive impairment. In the UK in 2019, approximately 900 000 people were living with dementia (mild, moderate and severe) with this figure projected to increase by 80% to approximately 1.6 million by 2040.^1^ This stark increase in dementia cases will bring a significant economic (eg, healthcare and social care) and societal (eg, unpaid care from friends/relatives) burden. Annual costs have been estimated at £94.1 billion in 2040, which will be a significant increase from £34.7 billion in 2019.^1^ Globally, the estimated costs of dementia are projected to rise to US$2 trillion annually by 2030.^2^ These figures highlight the urgent need for the development and implementation of effective prevention strategies and policies to target the established modifiable risk factors of dementia.^3 4^

In 2020, the Lancet Commission highlighted 12 modifiable risk factors that account for around 40% of dementias worldwide, which could theoretically be prevented or delayed.^4^ Of the 12 modifiable risk factors for dementia, seven could be positively influenced through the provision of healthy urban environments (namely, hypertension, obesity, depression, social isolation, physical inactivity, diabetes and air pollution). Healthy urban environments have the potential to provide population level benefits.^3^

The design of the urban environment could both negatively and positively impact cognitive health. For example, poor-quality, high-density, urban environments are associated with potentially harmful levels of traffic, air and noise pollution and environmental degradation, all of which have adverse effects on human health.^5^,^8^ A recent systematic review reported that higher levels of ambient air pollution were consistently associated with lower levels of global cognitive function, while extended exposure to high levels of particulate matter smaller than 2.5 micrometres (PM_2.5_) was associated with accelerated cognitive decline in several longitudinal studies. PM_2.5_ levels were associated with higher dementia incidence across several studies.^9 10^ Conversely, certain urban design features such as walkable and cycle friendly environments, mixed land use, access to green space and public spaces, proximity to community resources and access to public transport have the capacity to reduce car dependency, promote active transport and physical activity, and, as a consequence, better cognitive health outcomes.^311^,^13^ These urban environment factors have the potential to facilitate mechanisms such as reductions in noise and air pollution, less sedentary behaviour and increased levels of physical activity which improves cardiometabolic health, reduces risk of obesity and reduces inflammatory markers and oxidative stress.^311^,^13^ For example, a systematic review and meta-analysis of longitudinal studies reported that physical activity was associated with an 18% reduction in dementia risk.^14^

Healthy urban environments can also support social activity including social interactions which are important for preventing dementia.^4^ Conversely, urban environments which offer few opportunities for social connections, social interactions and sociable behaviours negatively impact cognitive health and accelerate cognitive decline.^15^ Meanwhile, many attributes of the social environment (such as crime and safety, social disadvantage, social capital, disorder and civic participation) predict both positive and negative outcomes for cognitive health.^16^ The social environment encompasses social relationships,^17^ which are also associated with dementia risk.^18^ Supportive social environments also offer contexts for provision of social support and social interaction, both of which are also associated with dementia.^18^,^20^ Livingston and colleagues noted the potential for an 8% reduction in the risk of dementia if social isolation in later life were to be eliminated.^4^ Therefore, it is important that urban environments are designed to support social activity.

However, to date, the literature reports inconsistent associations between cognitive health outcomes and environmental exposome factors. This may be because elements of the environmental exposome are being studied in isolation, with little regard for the complex interrelationships between environmental variables.^21^ This may also be relevant for physical activity particularly in the urban environment, where other features of urban design bring with them potential negative effects on biomarkers and cognitive health.^22^

The evidence base is somewhat lacking regarding the provision of supportive urban environments and their role in direct and indirect mechanisms operating in preventative pathways that could reduce the risk of mild cognitive impairment and dementia.^3^ Establishing and quantifying the direct and indirect mechanistic pathways that relate to the urban environment and the risk of mild cognitive impairment and dementia is critical. Such mechanisms may invoke a variety of factors that are part of the urban environmental exposome (eg, road networks, access and quality of green and blue space, public transport infrastructure and pollution), the social environment (eg, crime, safety, social disadvantage, civic participation) and lifestyle behaviours (eg, physical activity); and so we need to establish how they operate and influence the risk of cognitive decline and dementia. Such knowledge can inform the design and development of evidence-based preventative strategies and policies.

The aim of this project is to investigate the impacts and possible mechanistic pathways of the urban environmental exposome on healthy ageing and cognitive health outcomes through the novel integration of multiomics, health behaviour and environmental exposures from urban environments. This could ensure the creation of healthy active places that are supportive, attractive and accessible to people as they age. A key objective is to use granular measures of environmental exposures and physical activity to investigate the plausible mechanistic pathways by which the urban environmental exposome influences cognitive health outcomes in older adults. This involves the collection and integration of five data types: (1) device-measured physical activity behaviour using accelerometery; (2) environmental exposure data using Global Positioning System (GPS); (3) geospatial environmental data using Geographic Information System (GIS); (4) molecular data and (5) a range of cognitive health outcomes. The integration of these data will enable the creation of high-resolution spatial datasets, detailing exposures to urban environment factors, such as green/blue spaces, air pollution, noise pollution and light pollution. This will facilitate the investigation of how physical activity may mediate the influence of the urban environment on cognitive health.

This protocol paper provides a detailed overview of the methodological approach to integrating accelerometry, GPS, GIS, molecular and cognitive health outcomes data to investigate the influence of the urban environmental exposome on cognitive health outcomes in older adults.

## Methods and analysis

We have followed the Strengthening the Reporting of Observational Studies in Epidemiology guidelines where appropriate.^23^

### Study design

A longitudinal study design will be implemented with attempts made to collect data for participants over a period approximately every 2–3 years (online supplemental figure 1). Wave 1 was implemented 2022–2023 with the implementation of wave 2 proposed for 2025/2026.

### Study population

For the purposes of the current study, we leverage the Northern Ireland Cohort for the Longitudinal Study of Ageing (NICOLA) and the NICOLA Harmonised Cognitive Assessment Protocol (HCAP) substudy (NICOLA-HCAP).^24^,^27^ NICOLA is a prospective representative cohort of over 8000 non-institutionalised individuals living across Northern Ireland, UK ≥50 years of age.^24 25^ Wave 1 of the NICOLA-HCAP study recruits a subsample of 1000 participants from NICOLA aged ≥65 years who previously took part in Wave 2 of NICOLA.^27^ The NICOLA-HCAP study is part of a framework for enhancing cross-national research within a group of ongoing studies worldwide, all of which are centred on the United States-based Health and Retirement (HRS) international family of longitudinal ageing cohort studies.^26 28^

The NICOLA-HCAP protocol consists of a battery of neuropsychological tests and is designed to provide detailed data on ability across a range of cognitive domains, including memory, executive function, language and visuospatial ability. The protocol was originally developed and implemented within HRS and has since been implemented in several other national ageing cohorts.^28^,^31^ The protocol is designed to be delivered in a harmonised way across studies to facilitate cross-national comparisons of dementia prevalence and risk factors. For the first wave of this study, we will recruit a sub-sample of participants from NICOLA-HCAP.

#### Sample size

The NICOLA-HCAP assessment will be offered to the NICOLA participants aged ≥65 years, with a random sample of 50% of those living alone (n=1695) and a random sample of 50% of those living in a partnership or with a sibling etc. (n=2401). The response rate for the NICOLA-HCAP is expected to be approximately 80% and assuming a selection rate of ~1 in 3 to achieve the target of 1000 participants. Of 1000 NICOLA-HCAP participants, there is an anticipated uptake to the current study of ~40% (n=400).

#### Participant recruitment

**Figure 1:**
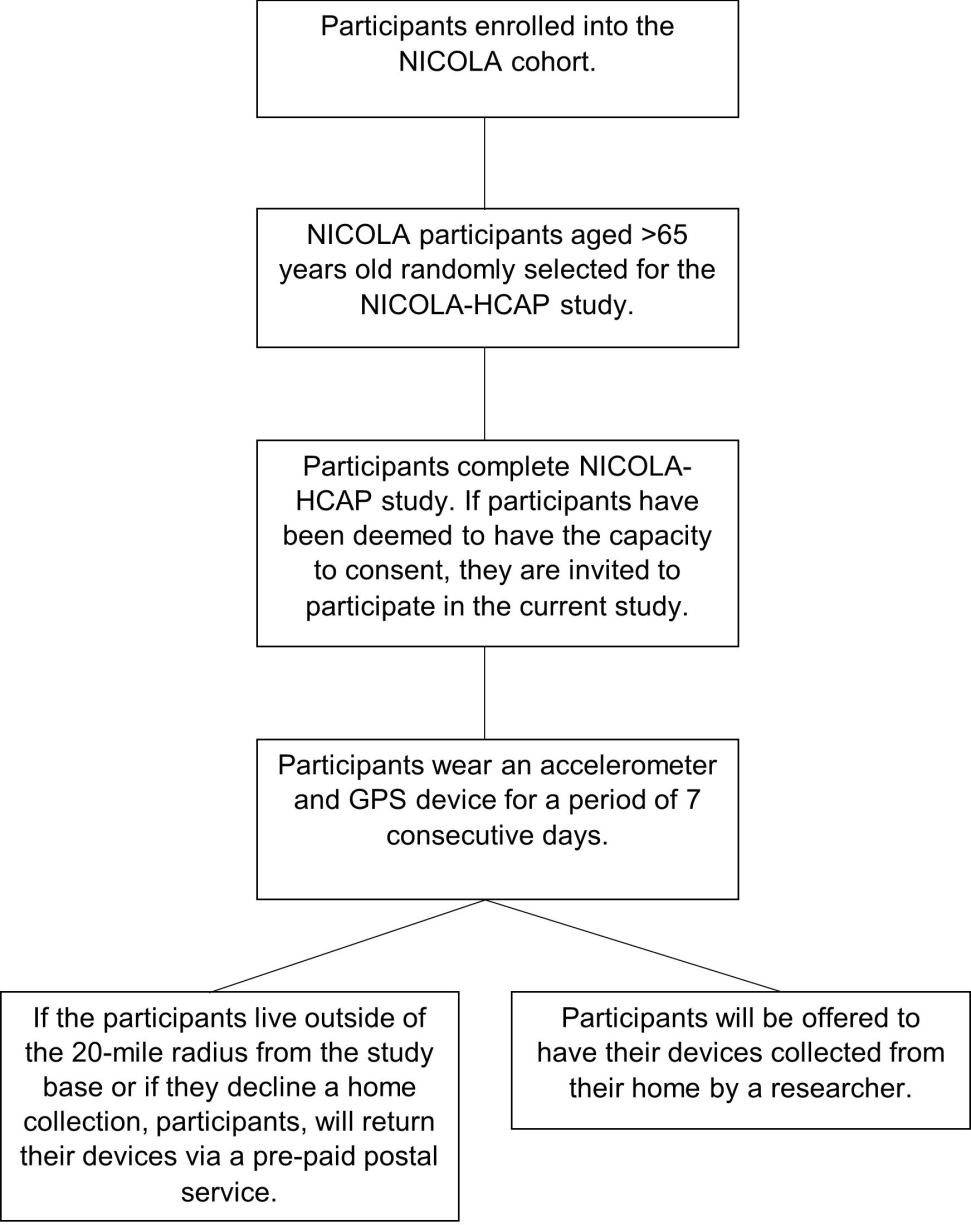
Flowchart depicting the study procedure. NICOLA, Northern Ireland Cohort for the Longitudinal Study of Ageing; NICOLA-HCAP: Northern Ireland Cohort for the Longitudinal Study of Ageing-Harmonised Cognitive Assessment Protocol.

Once recruited to the NICOLA-HCAP study, participants will be visited in their home by a trained NICOLA-HCAP researcher.^27^ Prior to commencing the cognitive health assessments, the researcher will determine if the individual has the capacity to consent. In the case of NICOLA-HCAP, this refers to a participant’s informed decision to participate in the completion of a battery of neuropsychological assessments. The researcher will judge if the participant can make a decision about participating in the research by considering their ability to: (1) understand the information relevant to the decision to participate or not; (2) retain the information related to the decision to be made; (3) appreciate the relevance of that information and weigh the information as part of the process of making the decision about participation and (4) communicate that decision to the researcher. If the participant does not have the capacity to consent to the researcher, they will not be eligible to participate in the current study. Recruitment commenced in February 2022 and will run for approximately 24 months (figure 1).^27^

##### Participant inclusion criteria

1. Have agreed to participate in the NICOLA-HCAP study.
2. Determined by the trained NICOLA-HCAP researcher that the individual has the capacity to consent.
3. The participant is willing to participate in the current study (in the form of providing written informed consent).

##### Participant exclusion criteria

1. Unable to complete the written NICOLA-HCAP assessment.
2. Not fluent in English.

### Study procedure

1. After completing the battery of cognitive tests, NICOLA-HCAP participants will be invited to participate in the current study by the NICOLA-HCAP researcher if they meet the inclusion criteria detailed above.
2. A short description of the current study and what is expected of potential participants will be verbally provided by the NICOLA-HCAP researcher, and participants will be provided with a paper copy of the participant information sheet and participant invitation letter.
3. Potential participants will have the opportunity to ask the NICOLA-HCAP researcher questions relating to any element of the current study.
4. At this stage, if participants verbally agree to participate in the current study, the NICOLA-HCAP researcher will provide them with a study pack. This pack contains two consent forms, two monitors (an accelerometer and a GPS device on an elasticated waist band), a monitor instruction sheet and a GPS charger.
5. If the potential participant requires more time to consider their participation, the NICOLA-HCAP researcher will only leave a study pack if the participant agrees that this is acceptable. It should be noted that leaving a study pack does not mean that the individual must participate in the current study, they are free to refuse participation or to withdraw at any time without providing a reason.
6. Participants will have the opportunity to read through the information and to familiarise themselves with the project before a project researcher contacts them via telephone approximately 1–3 days after their NICOLA-HCAP visit. This call aims to: (1) ensure that the participants understand the procedures; (2) provide participants with an opportunity to ask questions and (3) ensure that a standardised measurement approach is undertaken. If at this stage the participant wishes to withdraw, they are free to do so, and the researcher will arrange collection of their study pack. If they are willing to participate, they will be asked to wear both monitors on the elasticated waist belt for seven consecutive days.
7. If a participant did not agree to a study pack being left at their home but they did agree to a researcher phoning to discuss their potential participation, the researcher will make contact via telephone 1–3 days following the NICOLA-HCAP study. If at this stage the participant agrees to participate, a researcher will arrange a time to drop/post a study pack to their home.
8. During the 7-day monitoring period, participants will be asked to wear the monitors on an elasticated belt during waking hours (ie, remove to go to bed). The devices may also be removed when undertaking any water-based activities. To reduce participant burden, the research team will only contact participants once by telephone to ensure that they are not having any issues and to answer any questions that they might have. The participants will also have been provided with a telephone number to enable them to contact the research team on with any queries they may have.
9. Following the 7-day monitoring period, the research team will contact the participant to arrange a date/time to collect the monitors along with one signed consent form from the participant’s home.
10. Alternatively, if the participant would prefer to have a postal return the research team will provide a pre-paid return package.

Please refer to online supplemental figure 1.

### Data collection and processing

#### Environmental exposures

##### GIS and environmental variables

The cohort data will be linked to geospatial data of urban environmental factors. This will include GIS variables (eg, housing density, land use mix, walkability), remote sensing, soil tracer data, and modelled noise and air pollution data. These linked data will enable the quantification of characteristics of the urban environmental exposome that are plausibly associated with cognitive health outcomes and the extent to which these associations can be explained by lifestyle behaviours (eg, physical activity, social activity) and physiological markers (eg, multiomics data), thereby ‘unlocking the power of location’.^32^

**Figure 2:**
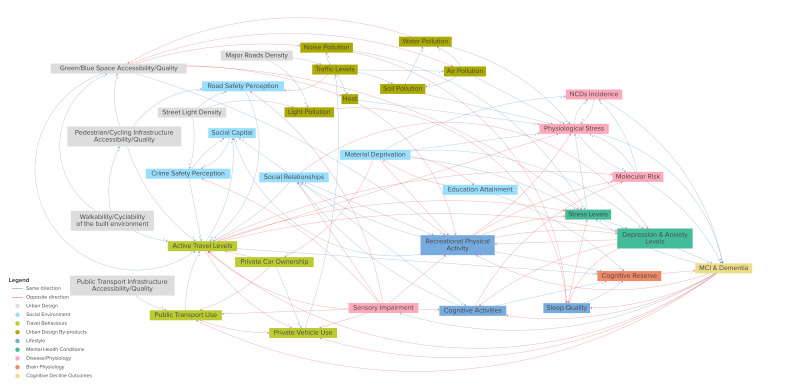
Diagram depicting the high level urban environmental exposome factors related to cognitive decline and dementia derived from the CLD (https://kumu.io/space-cld/space-cld). CLD, casual loop diagram.

The choice of variables has been informed by our previous systematic review of the built environment correlates of physical activity in older adults,^33^ and supplemented by in-depth focus groups with 33 older people (mean age 71 years old) conducted in the HULAP study.^34^ In addition, Group Model Building workshops were conducted with the research team to develop a casual loop diagram (CLD).^35^ The CLD identified established and hypothesised urban environment, lifestyle, health and physiological determinants of cognitive decline in older adults, and the dynamic interrelations between these factors. The CLD identified 10 domains, including urban design, social environment, travel behaviours, environmental by-products (eg, air pollution), lifestyle factors, mental health, disease/physiology, exogenous factors and cognitive decline outcomes (figure 2; https://kumu.io/space-cld/space-cld). The CLD informed our consideration of which urban environmental exposome factors to take forward in our analyses for plausible mechanistic pathways.

We will establish objectively measured walkability indices (based on density, land use mix, connectivity metrics for the 500 m, 800 m and 1000 m hinterlands of the study population (table 1). In addition, to area-level modelled air pollution (eg, PM2.5 and NO2), we will use urban soil data which can act as urbanisation tracers of atmospheric and traffic pollution (eg, arsenic (As), molybdenum (Mo), tin (Sn), antimony (Sb) and lead (Pb)).^36 37^ Studies have shown that ultrafine particles of these environmental toxins may become blood-borne and translocate to other tissues such as the brain.^38^ Synthesis and analysis of the datasets, including the exploration of the derivation of a novel ‘urban environment score’, will identify facilitating/impeding environment features for cognitive health and physical activity of older adults.

**Table 1:** Geographical information system data sources

Densification	Housing density; population density using Northern Ireland Statistics and Research Agency (NISRA) Census and Geographic Information System (GIS) metrics from Land & Property Services (LPS).
Infrastructure	Walkability indices from LPS (based on density, land use mix, connectivity, retail plot ratio) for the 500 m/1000 m; Bicycle lanes and trails; pavements; greenways.
Land use	Land use mix; land use type (area and distance to commercial, residential, agricultural, industrial, transport, hospital/medical, educational, parks, green/open space, recreational, ‘other land use’) from LPS/OSNI; land use accessibility; land use density and land use pattern.
Natural environment	Normalised Difference Vegetation Index (NDVI) (already curated from remote sensing data); access to green space and blue space (water) from UK Land Cover Map (Centre for Ecology and Hydrology).
Transportation	Road line, bus stop and train station densities (proxies for air and noise pollution) from LPS and Translink; road speed and traffic collisions from the Police Service NI; car ownership and travel mode from NICOLA; car ownership from Dept Infrastructure surveys and census indicate local environmental conditions.
Air, noise and light pollution	Estimates of potential exposure to ambient air pollution (NO2 and PM2.5) from DEFRA NiOS reports, and noise pollution for the Belfast A and M road network and data from Belfast City Council; Light at Night
Soil geochemistry	Urban geochemical data (1164 soil samples; Geochemical analysis for 58 elements; sampling density 4 sites per sq km) as part of the Tellus Survey; and 48 soil urbanisation tracers (Co, V, Cr, Ni, Zn, Sn, Pb, Sb, As and Mo).
Social environment	Social capital; social networks; social support; UCLA Loneliness Scale from NICOLA (Wave 1 and Wave 2); area-level crime; social disorder and social disadvantage from NI Statistics and Research Agency.
Other	Slope; neighbourhood theme/patterns; aesthetics and safety.

We will spatially join the cohort data to the georeferenced environmental data using the GPS x,y coordinate data. ArcGIS Pro software will be used to prepare the GIS data.^39^ GIS tools, including spatial joins, spatial summary statistics, density analysis and network analysis will be used to investigate how the cohort interacts with urban environmental exposome factors. Linear buffers of different sizes at 100 m intervals up to 1200 m around participants’ home addresses will be joined spatially to the environmental exposome data. In addition, the circular buffer sizes within the data are 100 m, 200 m, 300 m, 400 m 500 m, 600 m, 800 m, 1000 m and 1200 m. We will create aggregated physical activity metrics with place definitions. This will be done using GPS coordinates recorded over 15 s intervals for 7 days for participants. Further, linear buffers of different sizes around road networks will be spatially joined to the environmental pollution data, integrating data around the road network. Linear road buffers for A and M category roads across all Northern Ireland have been derived at 300 m, 500 m, 1000 m and 2000 m.^40^ We derive variables that measure the environmental factors within these road buffers. This will provide the sample size within the cohort within different proximities to road networks to be identified for further analysis. A variety of statistical analyses and spatial data analysis approaches will be used to investigate the relationship between the cohort activities, cognitive health outcomes and the urban environmental exposome (see later).

### Physical activity and sedentary behaviour (accelerometry) mediating and exposure variables

Participants will be asked to wear an ActiGraph GT3X or a GT3XP-BTLE accelerometer on the right hip for seven consecutive days to measure physical activity (ie, light, moderate and vigorous) and sedentary (eg, amount of time spent sitting) behaviour. Following wear, the raw accelerometry data will be downloaded, screened, cleaned and processed using ActiLife 6 Software (ActiGraph, Pensacola, Florida, USA). Accelerometer data will be collected at 100 Hz, and processed data will be stored as ‘activity counts’ per 15 s epochs. The activity intensity of each 15 s epoch will be classified using the Copeland & Esliger cut points (sedentary behaviour ≤99, light physical activity 100–1040 and moderate-to vigorous physical activity ≥1040).^41^ To be included, a participant needs at least 3–5 days of at least 8–10 hours of wear time. Non-wear is defined as a period of 120 min.^42^ The data will be scored, and specific cut-points will be applied as follows: sedentary behaviour ≤99; light physical activity 100–1040 and moderate-to vigorous physical activity ≥1040.^41^

Derived physical activity variables from accelerometry will include:

- Average acceleration (activity counts).
- Average acceleration per day.
- Average acceleration per weekday.
- Average acceleration per weekend day.
- The most active part of the day.
- Average acceleration in the most active hours.
- The least active part of the day.
- Average acceleration in the least active hours.
- Time spent in sedentary behaviour.
- Time spent in light physical activity.
- Time spent in moderate physical activity.
- Time spent in vigorous physical activity.

### GPS

Participants will also be asked to wear a GPS device (Qstarz BT-Q1000XT) concurrently with the accelerometer for seven consecutive days (the exact placement of the GPS device (eg, right, or left hip) does not influence the recording). The GPS device is a ‘black box’ monitor that uses GPS technology to record the location of participants. Although the data are recorded in real time, the collected data cannot be accessed by the research team until the participant returns the device, therefore preserving anonymity of real time location. The GPS monitor will provide an x.y coordinate for every 15 s. This allows physical activity data from the accelerometer to be accompanied by locational information by matching the timestamps from each device. Although we will have access to the x.y coordinates of participants movements, and therefore an understanding of home and neighbourhood locations, data will be analysed by assigning each x.y coordinate of the participant home address a land use category (eg, home, parcel, greenspace) and subsequently converted into variables that represent the total time spent in specific land use. GPS data will be downloaded using QTravel software.

### GIS, accelerometry and GPS data integration—exposure through behaviour

**Figure 3:**
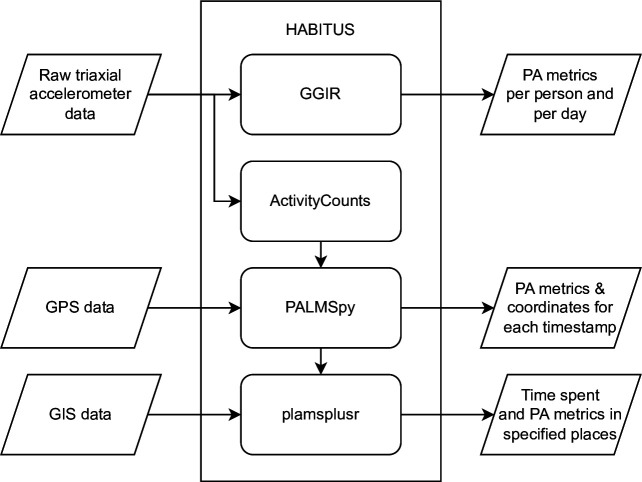
Data integration process using HABITUS. GIS, Geographic Information System; GPS, Global Positioning System; HABITUS, Human Activity Behaviour Identification tool and data Unification System.

We will use the Human Activity Behaviour Identification tool and data Unification System (HABITUS) to process and integrate the accelerometer, GIS, and GPS data.^43^Available as an R package,^44^ HABITUS streamlines the data processing in four other existing tools (GGIR, ActivityCounts, PALMSpy and palmsplusr). Figure 1 shows the data integration process through these tools. HABITUS also ensures autocalibration and imputation of missing data for accelerometer and GPS recordings. As both the accelerometer and GPS devices were set to record every 15 s, this enables the physical activity behaviour data from the accelerometer to be linked to the environmental location data (ie, x.y coordinates) from the GPS device by timestamp. These integrated data will be merged using an ‘exposure through behaviour’ method, which has been outlined elsewhere.^45^ In brief, the timestamps from both the accelerometer and GPS data will be matched, so that time spent in every physical activity intensity (ie, sedentary behaviour and light, moderate and vigorous physical activity) or travel mode (ie, active or passive), in every pre-determined space (eg, home, parcel, local neighbourhood and green and blue space) via GIS analysis can be calculated. See figure 3 for schematic diagram of data integration.

Derived variables following data integration via HABITUS will include:

- Time spent in/out home, parcel, neighbourhood (500 m and 1000 m).
- Time spent in green and blue spaces.
- Time spent in non-domestic properties (eg, shops, offices, warehouses, licensed premises, sporting recreation, community centres).
- Time spent in urban or rural areas.
- Time spent near or on road networks.
- Time spent in different levels of air, noise, light pollution.
- Time spent in different transportation modes (active and passive).

Additionally, we will select a subset of GPS coordinates that belong to trips outside of the home location. Using this subset, we will identify a minimum convex hull, a polygon enclosing all points, for each participant. This polygon will then be used to define each participant’s ‘activity space’ and overlay this polygon with corresponding GIS data to derive additional variables.^46^

Derived variables based on activity spaces will include:

- Average exposure to air pollution outdoors.
- Average exposure to noise pollution.
- Average exposure to light pollution at night.
- Visits to non-domestic properties (eg, shops, offices, warehouses, licensed premises, sporting recreation, community centres).

#### Molecular mediating data

Blood-derived biochemical data is available for 28 markers for 3082 participants within the NICOLA cohort (see online supplemental table 1). Blood-derived biochemical, genetic and epigenetic data will be combined with demographic, clinical, behavioural and environmental data for multimodal analyses. DNA was extracted from buffy coats by Eurofins Scientific, quantified using PicoGreen, normalised and aliquoted into multiple working volumes in cryvials to minimise freeze-thaw cycles and maximise the quality of DNA. Genotype data was generated on the Infinium CoreExome-24 array (Illumina, USA), with standard quality control applied and imputation to the 1KGP3, HRC and TopMed reference panels. Kinship matrices have been generated. Association analysis will be performed, but the primary utility of genetic data is to enable Mendelian Randomisation to explore causal inference and to help interpret epigenetic findings. DNA methylation is the primary molecular ‘ome’ of interest for this study, due to its ability to dynamically changes in response to medical, lifestyle and environmental changes. Methylation data were generated using Infinium MethylationEPIC 1.0 BeadChips (Illumina, USA) after bisulphite treatment with the EX Zymo Methylation Kit (Zymo Research, USA) following manufacturer’s instructions. Participant samples were randomly distributed across 249 arrays, with quality control including eight duplicate samples (n=16) and evaluation of the bisulphite treatment conversion efficiency, dye specificity, hybridisation and staining. Samples were initially assessed using GenomeStudio v2011 and the BeadArray Controls Reporter software platforms (Illumina, USA). Crossreactive probes, those located within 3 bp of common SNPs, and those on sex chromosomes will be excluded from primary analyses. Unreliable probes and samples will be removed before association analysis is performed. Proportional white cell counts will be estimated for CD8+T, CD4+T and CD19+B lymphocytes, CD56+ natural killer (NK) cells, CD14+ monocytes and CD15+ granulocytes using the Houseman method.^47^ Beta values were generated before M values were derived for all sites. P values were computed using the limma method for each site.^48^ Hierarchical linear models from the limma package were employed and fitted using Bayes approach on the derived M values. P values were generated for each of the four differential analyses conducted. RnBeads will be used to perform EWAS with single site resolution with multivariate regression analysis performed for quantitative traits adjusting for sex and age. RNA was collected in Paxgene tubes and extracted by Eurofins with quantitation performed on a Bioanalyser. Samples with a RIN >6 underwent sequencing using either a whole transcription or AmpliSeq technology for library preparation on an Ion Chef and subsequent sequencing on an Ion Proton or Ion GeneStudio S5 system (Life Technologies, USA). Transcriptome-wide association analysis will be performed, but the primary utility of this dataset for this project is to help understand the functional impact of epigenetic changes. Individual ‘omic’ analyses, exploring contributions from individual omics as well as a combined model, will be conducted with Mendelian Randomisation employed to explore causal influences with a composite multimodal biomarker signature generated. This project will provide more knowledge on the mechanistic pathways underlying how the urban environmental exposome affects cognitive outcomes in older adults, identify which environmental impact has the most biological impact, and has potential to identify a multiomic signature for people at higher risk of cognitive decline.

#### Cognitive health outcome variables (HCAP)

Cognitive health will be assessed by the HCAP, which comprises 19 measures of cognitive health which after following written informed consent will be conducted by a trained researcher in the participant’s home (table 2).^27^

**Table 2:** HCAP measures of cognitive health^26 27^

Outcome measure	Cognitive domain
Mini-Mental State Examination (MMSE)	Global cognitive status
HRS Telephone Interview for Cognitive Status (HRS-TICS)	Global cognitive status
CERAD Word List Learning and Recall—Immediate	Episodic memory (immediate)
Retrieval fluency (Animal naming)	Language/fluency
Letter cancellation	Attention/processing speed
Backward count	Attention/processing speed
10/66 Respondent	Global cognitive status
CERAD Word List Recall—Delayed	Episodic memory (delayed)
Story recall—Immediate	Episodic memory (immediate)
CERAD Word List—Recognition	Episodic memory (recognition)
CERAD Constructional Praxis—Immediate	Visuospatial ability
Symbol-Digit Modalities Test	Attention/processing speed
CERAD Constructional Praxis—Delayed	Visual memory (delayed)
Story recall—Delayed	Episodic memory (delayed)
Story recall—Recognition	Episodic memory (recognition)
HRS Number Series	Executive function
Raven’s Standard Progressive Matrices	Abstract reasoning
Trail Making Test (Part A & B)	Executive function/attention/processing speed
CES-D Depressive Symptoms	Assessment of presence and level of depression

Tests listed in order of administration. Psychological Assessment Resources (PAR), 16 204 North Florida Avenue, Lutz, Florida 33549, from the Mini Mental State Examination, by Marshal Folstein and Susan Folstein, Copyright 1975, 1998, 2001 by Mini Mental LLC. Published 2001 by PAR. [Copyright@parinc.com].

CERAD, Consortium to Establish a Registry for Alzheimer’s Disease; CES-D, Centre for Epidemiological Studies Depression Scale; HRS, Health and Retirement Study.

NICOLA-HCAP also includes an informant interview whereby the participant is asked to nominate a family member or friend to answer questions about their daily activities and cognitive abilities over time (table 3). The interview includes family or friend demographics and the following six measures:

**Table 3:** HCAP informant interview measures^26 27^

Outcome measure	Description
Family or friend demographics	Demographic profile of informant
Jorm IQCODE	Assessment of cognitive decline
Blessed dementia rating scale—Part 2	Assessment of ability to do basic self-care activities
HRS Activities questionnaire	Assessment of participant activity engagement
CSI-D—Cognitive Activities Questionnaire	Assessment of participant’s cognitive activity engagement and ability
10/66 Informant Questionnaire	Assessment of ability to do daily activities
Blessed dementia rating scale—Part 1	Assessment of additional activities and mental ability

Tests listed in order of administration.

CSI-D, Community Screening Instrument for Dementia; HRS, Health and Retirement study; IQCODE, Informant Questionnaire on Cognitive Decline in the Elderly.

Following data collection and processing, domain-level factor scores derived from the scores on the cognitive battery will be used in conjunction with informant measures to develop algorithm-based research diagnoses of probable dementia or cognitive impairment. This approach has previously been used to estimate dementia prevalence in the HRS-HCAP substudy.^49^ In a supplementary analysis, we will retrofit this predicted algorithmic score to the full Wave 1 and Wave 2 NICOLA longitudinal dataset.

### Confounders

Confounders such as age, sex, education, employment status, smoking, diet, cardiovascular risk factors, depression, years at home address will be accounted for.

### Longitudinal follow-up

Participants will be followed up through a second wave of data collection in conjunction with the second wave of NICOLA-HCAP in 2026. Participation in wave 2 will be offered to any surviving members of the wave 1 sample who are not living in a nursing home, can provide written informed consent and have been deemed to have the capacity to consent. In addition, a random sample of new participants (≥65 years) who have been recruited to NICOLA and subsequently NICOLA-HCAP through a sample refresh and who meet the inclusion/exclusion criteria will also be invited to participate. The same study procedures outlined above will be followed. This will provide a longitudinal integrated dataset which includes: (1) physical activity behaviour; (2) environmental exposure data; (3) geospatial environmental data; (4) biological data and (5) a range of cognitive health outcomes. Longitudinal high-resolution spatial datasets will be created facilitating investigations into the influence of the urban environment on cognitive health over time. Wave 2 of this study and NICOLA-HCAP have been planned for 2025/2026, pending the successful obtainment of funding. Please see online supplemental figure 1.

### Statistical analysis

Below we provide an overview of three statistical approaches that we will apply to the integrated data.

#### Total effect analysis

**Figure 4:**
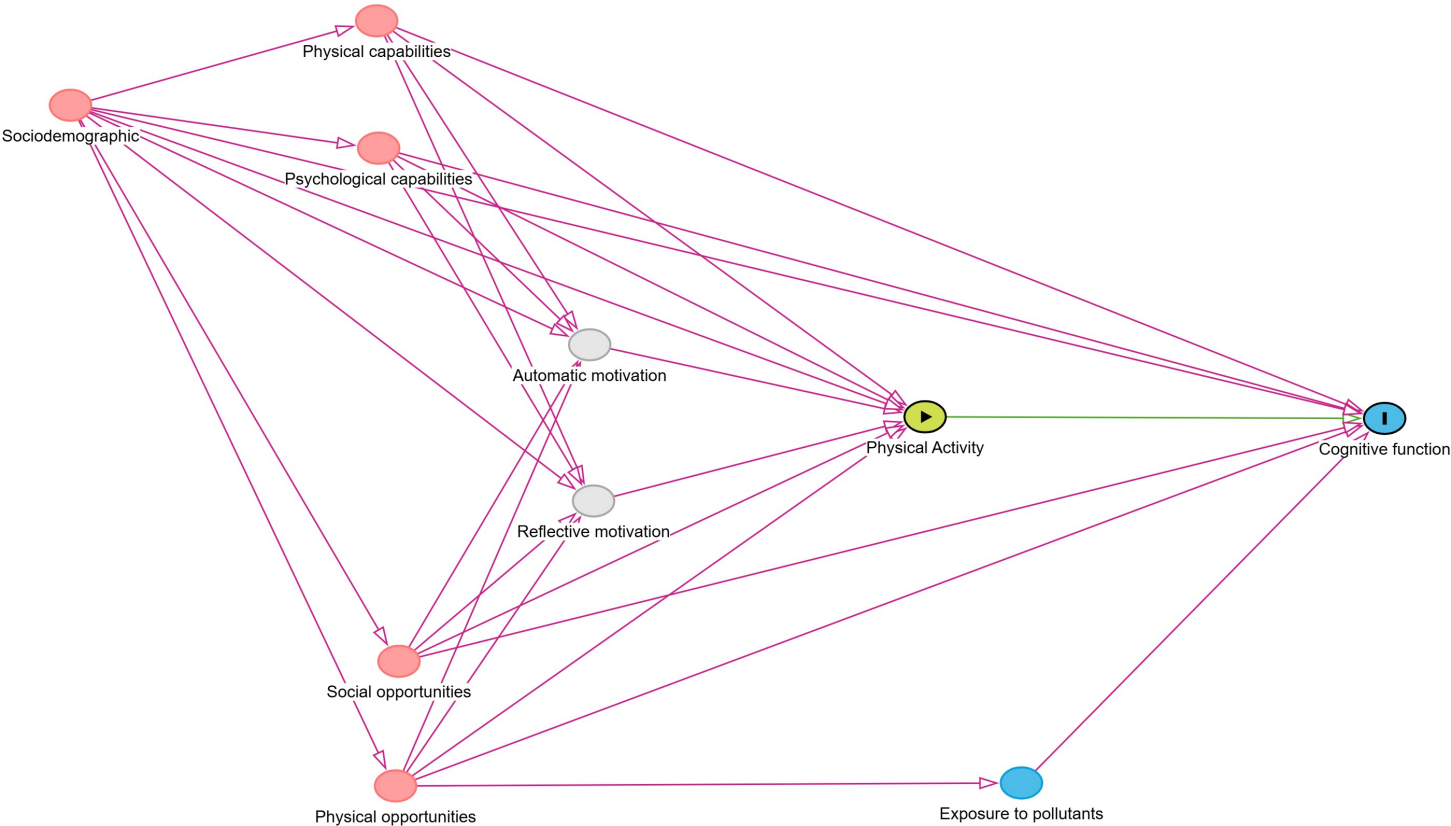
Preliminary directed acyclic graph for the effect of physical activity on cognitive function.

The focus of this analysis is the total effect of physical activity on cognitive function. The exposure variable will be operationalized as average daily MVPA minutes. We will assume that 7-day accelerometer measured physical activity represents the regular physical activity of a participant. The outcome variable will be operationalized as the MMSE score. We will use a directed acyclic graph (DAG) to identify our causal assumptions and a sufficient adjustment set according to d-separation rules.^50 51^ A preliminary DAG is provided in figure 4. We will employ the backdoor criterion for identification of the causal effect.^51^ This involves identifying potential confounding variables that could influence both the exposure and the outcome. Potential confounders include sociodemographic factors (age, sex, education, deprivation and marital status), capabilities (mobility, grip strength, sensory impairments) and opportunities (availability of greenspace and social relationships). By conditioning on these variables, we will be able to identify the total effect of physical activity (exposure) on cognitive function (outcome). Causes of the physical activity behaviour will be identified based on the COM-B model of behaviour.^52^ This will include physical (eg, physical or sensory impairments) and psychological capabilities (eg, previous cognitive function levels); physical (eg, access to recreation facilities) and social opportunities (eg, social support) of participants as well as sociodemographic characteristics. Multivariable linear regression models with doubly robust estimators will be used to estimate the average causal effect.^53^ As a sensitivity analysis, we will test various ways of operationalising the exposure and outcome. This will include average daily acceleration and average daily sedentary time for exposure, episodic memory and processing speed for outcome.

#### Mediation analysis

Associations of environmental attributes (GIS data) with lifestyle behaviours and cognitive health will be estimated using multilevel modelling in a structural equation modelling framework,^54^ which is able to account for administrative unit-level and person-level (repeated measures) clustering and is appropriate for data with different distributions.^55^ Contemporary guidelines on discerning general from specific context effects will be followed.^56^ Plausible confounders will be identified using a pre-specified conceptual framework in conjunction with rules governing appropriate covariate selection and depending on the mechanism proposed, will be modelled as either a time-non-varying covariate, moderating or mediating effect as appropriate.^57^ Measurement of cognitive function took place in Waves 1+2 of NICOLA as well as the HCAP (in 2021), (26) yielding data that is amenable to use in a latent change score mediation within a multilevel SEM framework, satisfying the temporal ordering assumption for mediation analyses.^54 58 59^ Current guidelines on reporting SEM analyses will be followed.^60^ As a sensitivity check, an alternative approach to mediation from the causal inference literature will be used to decompose the total effect of the environment on cognitive function into direct, indirect and interactive effects.^61^ Based on a bias-corrected bootstrapping test of mediation, with 2000 bootstrap samples, and assuming a small (simulated at 0.14) effect size for both the path from environment to mediator and from mediator to environment, a sample size of approximately 462 would be required for a power of 0.8.^62^ Complementary to this, to further inspect putative causal relations between mediators and cognitive function, a 2-wave latent change score model (as previously used)^63^ will be used on a subset of Wave 1 NICOLA and NICOLA-HCAP respectively. Traditional bias-corrected bootstrapping will be used to determine whether lifestyle behaviours mediate the association between environmental exposures and cognitive outcomes. We will employ two strategies to mitigate collinearity, either: (1) composite measures of collinear variables or (2) the residual error term from preliminary bivariate regressions, will be included in multiple predictor models. Missingness will be assessed and, if missing at random or completely at random, will be addressed by full information maximum likelihood estimation for the mediation analyses.

#### Compositional data analysis

To analyse the relative proportion of time spent in different activities, we will also investigate the association between movement behaviour (exposure), urban green and blue space (exposure) and cognitive health (outcome) by implementing a compositional data analysis approach. Having access to accelerometry, GPS and GIS enable investigations that are focused both behaviourally (part 1) and geographically (part 2).

Part 1—behaviour focused; will examine:

(1a) during waking hours, how is time spent in one movement behaviour relative to others (ie, sedentary behaviour, light physical activity and moderate-to-vigorous physical activity) and associated with cognitive health?

(1b) how does the risk of cognitive decline change, when the time spent in specific movement behaviours (ie, sedentary behaviour, light physical activity and moderate-to-vigorous physical activity) are redistributed across waking hours?

Part 2—addition of geographical location (green and blue space); will examine:

(2a) during waking hours how is time spent in one movement behaviour relative to others (ie, sedentary behaviour in green and blue space, sedentary behaviour everywhere else, light physical activity in green and blue space, light physical activity everywhere else, moderate-to-vigorous physical activity in green and blue space and moderate-to-vigorous physical activity everywhere else) and associated with cognitive health?

(2b) how does the risk of cognitive decline change, when the time spent in specific movement behaviours (ie, sedentary behaviour in green and blue space, sedentary behaviour everywhere else, light physical activity in green and blue space, light physical activity everywhere else, moderate-to-vigorous physical activity in green and blue space and moderate-to-vigorous physical activity everywhere else) are redistributed across waking hours?

Multivariate analysis of variance will be conducted to investigate the difference in participant compositions (ie, part 1a and 2a) and the outcome of cognitive health. Compositional data analysis which is based on isometric log-ratio data transformation will then be conducted to model the association of time spent in different movement behaviours during waking hours and cognitive health.^64 65^ Minute-by-minute isotemporal substitutions will be made from one behaviour to another while holding the remaining behaviours constant, to model change in cognitive health. Adjustments for potential confounders and mediators will be made and includes age, sex, socioeconomic status, lifestyle behaviours (eg, smoking, drinking), disability, car ownership etc.

To control for multiple comparisons, the false discovery rate correction will be applied using the Benjamini-Hochberg procedure, adjusting for the total number of analyses conducted.

### Patient and public involvement

To determine the acceptability and feasibility of this study we consulted with the NICOLA Healthy Ageing Research Advisory Group. This advisory group is made up of volunteers from AgeNI, a registered charity in Northern Ireland for older people and current NICOLA participants. Prior to commencing the study during the design and development phase the research team worked with members of the Advisory Group to obtain their feedback on a range of study elements. This included (but was not limited to): potential participant burden, monitor wear time, study procedures (eg, number participant phone calls, method of pack return) and future dissemination plans.

## Ethics and dissemination

###  Ethical approval

This study has been approved by the Queen’s University Belfast, Faculty of Medicine, Health, and Life Sciences Research Ethics Committee (MHLS 21_72). Prior to participation in the current study informed consent was obtained, with all participants completing and returning a written consent form.

### Data management

All researchers adhere to the Queen’s University Belfast quality assured process for data management, which follows the UK Economic and Social Research Council’s (ESRC) Research Data Policy. A Strategic Management Team (SMT) comprised of the experienced principal investigators and co-investigators, meets quarterly throughout this study. A monitoring schedule covering the roles and responsibilities of the researchers, the SMT for data quality, integrity, compliance, dissemination, safety, governance and ethics was developed and agreed. Data quality, follow-up and trial monitoring will be facilitated through a project specific database, including validation and compliance reports.

### Dissemination

This study protocol details an ongoing interdisciplinary research project that aims to examine the causal mechanisms between the urban environment and cognitive health, with a particular focus on the plausible pathways through physical activity, social activity and urban by-products in older adults in Northern Ireland. In addition, it has the potential to advance the methodological processes regarding the integration and analysis of accelerometry, GPS, GIS and cognitive health outcomes. Disseminating this work, which highlights the methodological approach for combining diverse datasets in a transparent and robust manner, allows future researchers to follow these methods to create and analyse granular temporospatial datasets using established methodologies. Regarding the findings from this study, they will be disseminated through peer-reviewed publications in high-ranking international journals, at national and international conferences and through the research teams far-reaching networks.

## Supplementary material

10.1136/bmjopen-2024-085318 online supplemental file 1
