# Supplementary material for: Integrating accelerometry, GPS, GIS and molecular data to investigate mechanistic pathways of the urban environmental exposome and cognitive outcomes in older adults: a longitudinal study protocol
Source: BMJ Open. 2024 Dec 10;14(12):e085318. doi: 10.1136/bmjopen-2024-085318 (PMC12182013; doi:10.1136/bmjopen-2024-085318)
Supplement: online supplemental file 1 [file bmjopen-14-12-s001.pdf]

Supplementary Figure 1. Study timeline.

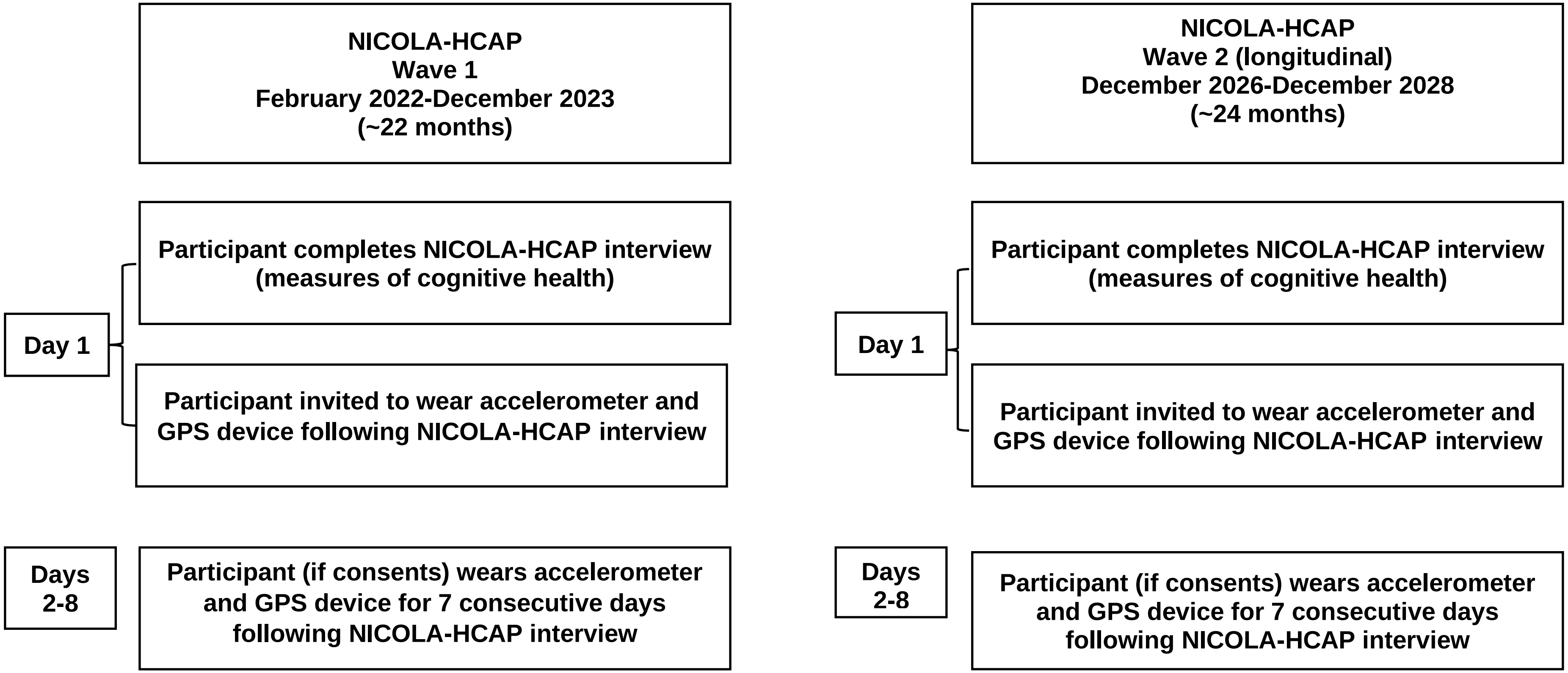

NICOLA-HCAP: Northern Ireland Cohort for the Longitudinal Study of Ageing-Harmonised Cognitive Assessment Protocol.

Supplementary Table 1. Blood-derived biochemical markers.

Blood-derived biochemical markers (n=28)
Alanine aminotransferase
Albumin
Alkaline Phosphatase
Apolipoprotein A
Apolipoprotein B
Aspartate aminotransferase
Calcium
Cholesterol
Creatinine
Cystatin C
Direct Bilirubin
Direct low-density lipoprotein
Gamma glutamyltransferase
Glucose
High density lipoprotein
High sensitivity C-reactive protein
Lipoprotein A
Oestradiol
Phosphate
Rheumatoid factor
Sex hormone binding globulin
Testosterone
Total Bilirubin
Total protein
Triglyceride
Urate
Urea
Vitamin D
