# Supplementary material for: Integrating accelerometry, GPS, GIS and molecular data to investigate mechanistic pathways of the urban environmental exposome and cognitive outcomes in older adults: a longitudinal study protocol
Source: BMJ Open. 2024 Dec 10;14(12):e085318. doi: 10.1136/bmjopen-2024-085318 (PMC12182013; doi:10.1136/bmjopen-2024-085318)
Supplement: Supplementary file 2 [file bmjopen-2024-085318.reviewer_comments.pdf]

PEER REVIEW HISTORY

BMJ Open publishes all reviews undertaken for accepted manuscripts. Reviewers are asked to complete a checklist review form and are provided with free text boxes to elaborate on their assessment. These free text comments are reproduced below.

ARTICLE DETAILS

Title (Provisional)

Integrating accelerometry, GPS, GIS, and molecular data to investigate mechanistic pathways of the urban environmental exposome and cognitive outcomes in older adults: a longitudinal study protocol.

Authors

Hunter, Ruth F.; Cleland, Claire; Trott, Mike; O’Neill, Sean; Küçükali, Hüseyin; Mullineaux, Shay; Kee, Frank; McKinley, Jennifer M; Neville, Charlotte; O'Hara, Leeanne; Marr, Calum; McAlinden, Michael; Ellis, Geraint; McKnight, Amy; Schipperijn, Jasper; McHugh Power, J; Duong, Trung; McGuinness, Bernadette

---

VERSION 1 - REVIEW

**Reviewer**	**1**
**Name**	**Halpin, Amy**
**Affiliation**	**The University of Maine**
**Date**	**23-Mar-2024**
**COI**	**N/A**

Comments to the Authors

This paper had a main goal of investigating how environmental, social, and physical factors interact to influence cognitive health outcomes in a community-based sample of Irish older adults.

The topic is interesting, and this is a necessary line of inquiry as thus far there is a paucity of literature published on the relationships between environmental factors and cognitive health. The manuscript was clear, theoretically sound, well thought out, and timely.

The study has several strengths; at the forefront is a robust study design, including consideration for understudied factors such as infrastructure, land use, and the natural environment as well as inclusion of a community-based sample with an eye toward generalizability of findings. Along these lines, I was impressed with their incorporation of ecological momentary assessments, which is lauded as being a more accurate assessment of pertinent study variables. I am also pleased to see a comprehensive neuropsychological assessment, though I do have some concerns in need of further clarification.

That said, I have provided some comments below to highlight some areas that the authors may consider addressing to strengthen the manuscript further and improve clarity prior to publication.

1) **Timeline**. It is unclear from reading through the current manuscript as to when the neuropsychological data will be collected? At the start of the study? After the 7 days? Longitudinally? Moreover, if multiple timepoints will be used to collect neuropsychological data, I suggest authors use alternate forms, particularly for memory measures. While the authors acknowledge a larger study when referring to the neuropsychological data, it would be clearer to the reader if the time points were clarified in this manuscript.

2) **Cognitive Screener**. Given the well established ceiling effects, and reduced sensitivity/specificity of the MMSE, I suggest the authors use a more fine-grained and robust cognitive screener. Ideas include the Montral Cognitive Assessment (MoCA) or the Addenbrooke's Cognitive Assessment (ACE-III).

3) **Neuropsychological Assessment**. I appreciate the range of domains evaluated by the different cognitive measures. However, I think it is important to add a measure of estimated premorbid intelligence given the well established relationship between education, cognitive reserve, and risk for cognitive decline. I also think it would behoove the authors to include a processing speed measure without motor demands such as WMS-III Mental Control, Oral Trail Making Test, or Oral symbol digit modalities to adjust for potential motor confounds such as arthritis or neuropathy. I'm also curious as to why the authors did not include a visual memory measure in their battery as a way to speak to potential lateralization of study findings.

4) **Statistical Analysis**. The authors propose robust statistical methods. However, given the number of analyses performed, a correction for multiple comparisons should be used. The authors may consider using an FDR correction, which often has a good balance between eliminating spurious findings and preserving actual relationships. Or, if findings are particularly strong, the authors may consider using a more conservative Bonferroni correction.

**Reviewer**	**2**
**Name**	**González-Caballero, Juan-Luis**
**Affiliation**	**The University of Cádiz, Statistics and Operations Research**
**Date**	**24-Mar-2024**
**COI**	**I have no conflict of interest**

Review of BMJ Open-2024-085318

First of all, I would like to thank the editors of the journal for the opportunity to review this project.

I am aware that a research project or protocol can be constructed in different ways, which can be correct as long as the fundamental sections that should be included in the project are properly stated.

In my opinion, the most important thing in a project is that once the hypotheses and objectives have been established, the materials and methods proposed must be very clear and appropriate for testing the hypotheses and evaluating the proposed objectives.

To achieve this, I believe that the first thing that would help the understanding of the project is to follow an appropriate order in the presentation of the materials and methods. Especially in projects where there are a large number of variables, such as this one. If this is not done, when reading the project one gets lost, and this has happened to me in this work. Therefore, the first thing I would ask of the authors is that they try to revise it in order to order it properly and increase its clarity.

In what follows, I will try to outline some aspects that I think it would be good to revise.

Abstract

In my opinion, the usual sections in the abstract of a research project are: Introduction, objectives, methods and key words.

- The first two sections are included in the "Introduction".

- Regarding the "Methods" section, it would be good to include a summary of the design, population and sample, variables and an outline of the analysis plan.

Additionally, a section on "expected results" could be included. I assume that the authors refer to this in the section entitled "Discussion".

Regarding the STRENGTHS AND LIMITATIONS of the project, I think this is not the place to include this section because it is difficult to understand when the reader has not yet examined the whole project. I think this section could be included before the ETHICS AND DISSEMINATION section.

Introduction

The main argument of the introduction is the convenience of analysing how environmental exposure in the urban environment and physical activity influence the cognitive health of individuals, based on the Lancet Commission report on the 12 modifiable risk factors for dementia.

For this purpose, the information provided by some studies in relation to the environmental setting and cognitive health is included. Results linking environments with problematic characteristics to cognitive health are also commented on.

Finally, the main objective of the project is established. To this end, five types of data are proposed to be analysed. The first three types seem reasonable, but in the case of the molecular data types it is not clear the rationale for introducing them, and how they will be integrated into the analysis. It is said that a multimodal analysis will be carried out. Could this type of analysis be better explained?

On the other hand, I understand that cognitive data are the outcome variables, but it is not clear to me why so many variables have to be collected. Will all of them be used? How?

Methods and analysis

Although the authors claim to follow the STROBE guidelines, I think there are some issues that could be clearer:

Study design

- In the "study design" section, the protocol talk about the objective, the cohort studies that will be used to conduct this protocol, how the first wave of this study will be recruited and the data that will be collected. I think that this section is not the place to give all these details. In my opinion they confuse the reader.
- I think this section should contain the type of design that is proposed for this study (what is it?), and depending on the design what groups there will be and if there will be follow-up. Having read the protocol several times, I am still not clear on these details.
- After the "study design" section, I would include a section (or several sections) on the target population, the accessible population and the study subjects: the sample, sampling technique, inclusion and exclusion criteria and recruitment. These sections should include the details found in the "study design" section.
- In the section on participant selection criteria, is it correct that there are no exclusion criteria?
- Regarding the proposed sample size, no statistical criteria are used for the precision of the estimates or the power of the contrasts to be used in the analysis. This seems to me to be very important to address, firstly because it would clarify the exposure and outcome variables to be handled in the project, and secondly because it will allow the feasibility of the project to be assessed. On the other hand, considering the number of exposure variables to be used and some types of analysis proposed on the multilevel SEM models, the size of 400 individuals seems to me to be a small sample size for the main objective of the protocol.
- The flowchart in figure 1 describing the recruitment process has a double branch at the end which is not clear.

Data collection and processing

- Regarding the groups of variables proposed to be used in the project, I think that an effort could be made to describe them in a clearer way. In addition, I have doubts that it is necessary to use so many variables for the objectives proposed in the project.
- The role of the three groups of variables related to accelerometer, GIS and GPS data seems clear. These data are proposed to be integrated using the HABITUS tool, but it is not clear which variables are obtained from this tool to introduce into the analysis.
- Regarding the molecular variables, it is not clear in my opinion what role they play in the protocol. For this type of variable, a multimodal analysis is proposed; is it possible to better clarify how this analysis will be carried out?
- Regarding the variables in the "Cognitive Health Outcomes" group, a large number of instruments (scales) are described to measure the cognitive health of the study participants, as well as other instruments for the informants. Are so many variables necessary to assess cognitive health, would the MMSE not be sufficient, and how to justify the other variables?

At the end of this section it is commented that "Following data collection and processing a research diagnosis of probable presence of absence of dementia and cognitive impairment will be ascertained using algorithms that will be developed in collaboration with colleagues from the Health and Retirement Study family of studies. In a supplementary analysis, we will retrofit this predicted algorithmic score to the full Wave 1 and Wave 2 NICOLA longitudinal dataset". This comment does not really explain what will be done with so many variables.

- I also think it would be more logical to have first described all the exposure variables (accelerometer, GIS and GPS), their integration, the molecular variables and finally the outcome variables on the cognitive health of the participants.
- Finally, I think it would be clarifying to emphasise at the end of the section which are the outcome variables (dependent) and which are the exposure variables (independent) and possible confounders, mediators and/or moderators.

Longitudinal follow up

In this section on follow-up, it is stated that a second wave of participants will be recruited using the NICOLA-HCAP study. It would be good to clarify:

- Is there any follow-up for the participants of the first wave?
- Is this second wave a second group of participants, what is its purpose in the study, and does it have any relation to the first wave?
- It is said that this second wave will be recruited with the same inclusion/exclusion criteria. What are the exclusion criteria? No such criteria are stated.

Statistical analysis

Regarding this section:

- In the first approach the total effect of physical activity on cognitive function is analysed, where it is indicated that for cognitive function the MMSE scale will be used. Will the other variables in table 2 and table 3 also be used?
- Regarding the exposure factors, MVPA will be used. Are these variables the ones obtained from the integration using HABITUS? Which specific variables are the exposure variables?
- It is stated that "Multivariable linear regression models with doubly robust estimators will be used to estimate the average causal effect", is this correct and is the dependent variable multivariate? On page 19, line 25 it says again "multivariate analysis of variance". What is the multivariate variable?
- On the other hand, isn't the model presented in figure 4 on the effect of physical activity on cognitive function a SEM model? And if so, aren't there variables in this figure that may act as confounders, mediators or moderators? Or perhaps this analysis is the one described in the next section "Mediation analysis"?

Finally, no rough timeline for the protocol is included.

VERSION 1 - AUTHOR RESPONSE

Reviewer: 1
Dr. Amy Halpin, The University of Maine

Comments to the Author:

This paper had a main goal of investigating how environmental, social, and physical factors interact to influence cognitive health outcomes in a community-based sample of Irish older adults.

The topic is interesting, and this is a necessary line of inquiry as thus far there is a paucity of literature published on the relationships between environmental factors and cognitive health. The manuscript was clear, theoretically sound, well thought out, and timely.

The study has several strengths; at the forefront is a robust study design, including consideration for understudied factors such as infrastructure, land use, and the natural environment as well as inclusion of a community-based sample with an eye toward generalizability of findings. Along these lines, I was impressed with their incorporation of ecological momentary assessments, which is lauded as being a more accurate assessment of pertinent study variables. I am also pleased to see a comprehensive neuropsychological assessment, though I do have some concerns in need of further clarification.

That said, I have provided some comments below to highlight some areas that the authors may consider addressing to strengthen the manuscript further and improve clarity prior to publication.

We thank you for taking the time to review our manuscript and we have now addressed each of your comments below.

1)Timeline. It is unclear from reading through the current manuscript as to when the neuropsychological data will be collected. At the start of the study? After the 7 days? Longitudinally? Moreover, if multiple timepoints will be used to collect neuropsychological data, I suggest authors use alternate forms, particularly for memory measures. While the authors acknowledge a larger study when referring to the neuropsychological data, it would be clearer to the reader if the time points were clarified in this manuscript.

We have taken this comment on board in addition to a similar comment made by Reviewer 2. In light of these comments, we have added a figure to the Supplementary material highlighting the study timeline. Please see Supplementary Figure 1 and pages 8 and 20.

We have also updated the Study Procedure section on page 7 to make clear that neuropsychological data will be collected prior to participants being invited to take part in the current study.

2) Cognitive Screener. Given the well established ceiling effects, and reduced sensitivity/specificity of the MMSE, I suggest the authors use a more fine-grained and robust cognitive screener. Ideas include the Montral Cognitive Assessment (MoCA) or the Addenbrooke's Cognitive Assessment (ACE-III).

We appreciate that there will likely be a ceiling effect with the MMSE and acknowledge the reduced sensitivity/specificity. However, the MMSE will not be used for screening purposes, and any investigation of possible dementia/MCI will be able to take advantage of the full battery of detailed cognitive tests, which will provide much more sensitivity overall.

Regarding the suggestion of using other tests, the battery of tests used in the NICOLA-HCAP sub-study is, by design, fixed to be harmonised with those used in the network of longitudinal ageing cohort studies centered around the US-based Health and Retirement Study. Details of test selection for HCAP are provided in Langa et al., 2020

(https://doi.org/10.1159/000503004); reasons for test selection include overlap with other studies, brevity and ease of administration within a larger battery, and harmonizability. We have added a brief section in the Study Population section on page 5 (see below) to make clear that the cognitive battery was not designed specifically for this study, but rather as part of an ongoing international harmonisation effort.

The NICOLA-HCAP protocol consists of a battery of neuropsychological tests and is designed to provide detailed data on ability across a range of cognitive domains, including memory, executive function, language and visuospatial ability. The protocol was originally developed and implemented within HRS and has since been implemented in several other national ageing cohorts (Lee et al., 2019; Mejia-Arango et al., 2020; Cadar et al., 2021; Bassil et al., 2022). The protocol is designed to be delivered in a harmonised way across studies to facilitate cross-national comparisons of dementia prevalence and risk factors.

3) Neuropsychological Assessment. I appreciate the range of domains evaluated by the different cognitive measures. However, I think it is important to add a measure of estimated premorbid intelligence given the well established relationship between education, cognitive reserve, and risk for cognitive decline. I also think it would behoove the authors to include a processing speed measure without motor demands such as WMS-III Mental Control, Oral Trail Making Test, or Oral symbol digit modalities to adjust for potential motor confounds such as arthritis or neuropathy. I'm also curious as to why the authors did not include a visual memory measure in their battery as a way to speak to potential lateralization of study findings.

We understand the reviewers concerns regarding the lack of premorbid measures or non-motor-based measures of speed. However, as explained above, the specific tests used are designed to be harmonised with the HCAP sub study conducted in HRS, and therefore the specific test used in the NICOLA-HCAP sub-study cannot be altered. The original selection of tests by HRS required balancing a broad range of tests with what was achievable within an assessment lasting around 1 hour, and as such not all possible tests could be included. However, as all participants have completed previous waves of the main NICOLA study, any future analyses will be able to take advantage of data previously collected, which includes cognitive assessments and measures of educational attainment.

The delayed recall part of the CERAD constructional Praxis test is a measure of visual memory; we have amended Table 2 to reflect this (see pages 14 and 15).

4) Statistical Analysis. The authors propose robust statistical methods. However, given the number of analyses performed, a correction for multiple comparisons should be used. The authors may consider using an FDR correction, which often has a good balance between eliminating spurious findings and preserving actual relationships. Or, if findings are particularly strong, the authors may consider using a more conservative Bonferroni correction.

Thank you for this suggestion. We have carefully considered our options, and added plans for a FDR correction for multiple comparisons at the end of the statistical analyses section (see page 23 and below).

To control for multiple comparisons, the False Discovery Rate (FDR) correction will be applied using the Benjamini-Hochberg procedure, adjusting for the total number of analyses conducted.

Reviewer: 2

Dr. Juan-Luis González-Caballero, The University of Cádiz
Comments to the Author:
Review of BMJ Open-2024-085318

First of all, I would like to thank the editors of the journal for the opportunity to review this project. I am aware that a research project or protocol can be constructed in different ways, which can be correct as long as the fundamental sections that should be included in the project are properly stated.

In my opinion, the most important thing in a project is that once the hypotheses and objectives have been established, the materials and methods proposed must be very clear and appropriate for testing the hypotheses and evaluating the proposed objectives.

To achieve this, I believe that the first thing that would help the understanding of the project is to follow an appropriate order in the presentation of the materials and methods. Especially in projects where there are a large number of variables, such as this one. If this is not done, when reading the project one gets lost, and this has happened to me in this work.

- **Therefore, the first thing I would ask of the authors is that they try to revise it in order to order it properly and increase its clarity.**

We thank reviewer 2 for their comments and we have responded to each comment below and amended the manuscript accordingly. Please see responses below.

In what follows, I will try to outline some aspects that I think it would be good to revise.

Abstract
In my opinion, the usual sections in the abstract of a research project are: Introduction, objectives, methods and key words.

-The first two sections are included in the "Introduction".

- Regarding the "Methods" section, it would be good to include a summary of the design, population and sample, variables and an outline of the analysis plan. Additionally, a section on "expected results" could be included. I assume that the authors refer to this in the section entitled "Discussion".

Regarding the STRENGTHS AND LIMITATIONS of the project, I think this is not the place to include this section because it is difficult to understand when the reader has not yet examined the whole project. I think this section could be included before the ETHICS AND DISSEMINATION section.

We have taken each of the comments above regarding the abstract and the strengths and limitations into consideration. We have followed the guidance of the journal Editor and the guidance provided on the journal website and updated both sections accordingly (see pages 1 and 2).

Introduction
The main argument of the introduction is the convenience of analysing how environmental exposure in the urban environment and physical activity influence the cognitive health of individuals, based on the Lancet Commission report on the 12 modifiable risk factors for dementia.

For this purpose, the information provided by some studies in relation to the environmental setting and cognitive health is included. Results linking environments with problematic characteristics to cognitive health are also commented on. Finally, the main objective of the project is established. To this end, five types of data are proposed to be analysed. The first three types seem reasonable, but in the case of the molecular data types it is not clear the rationale for introducing them, and how they will be integrated into the analysis. It is said that a multimodal analysis will be carried out. Could this type of analysis be better explained?

A core strength of this protocol is the direct measurement of a range of variables, including physical activity and environmental exposures, which will be analysed for their impact on cognitive health outcomes. Each person has both inherited and acquired biological factors that influence how they individually respond to exposures, making analysis of the underlying biology an integral part of this protocol. The molecular research described in this protocol will improve our biological understanding of a person's genetic risk of health outcomes and the way in which they respond to intrinsic and extrinsic factors. The genetic data also enable Mendelian Randomisation analysis to help identify which exposures are causally linked to cognitive outcomes compared to those that represent statistical associations not in the causal pathway. Some epigenetic features are inherited, but others respond dynamically in response to medical, lifestyle, and / or environmental variables. Epigenetic analysis will provide more information on the biological pathways underlying how exposures influence health outcomes, additionally providing an 'early warning' of detrimental biological changes. Another benefit to epigenetic analysis is the ability to develop DNA methylation profiles as surrogate markers of exposures, which is particularly relevant when extending to other studies that may not have the same in-depth exposure information as proposed for this study. RNA-seq analysis facilitates functional analysis of epigenetic changes that influence gene expression.

Multimodal analysis is simply a methodological approach to comprehensively analyse different types of data together to provide a more holistic understanding of complex systems. In this way we can build a picture over multiple layers including different data modalities, identifying risk factors and patterns that would be missed when analysing each type of data (modality) separately. This approach also helps to identify biological interactions and regulatory dependencies.

On the other hand, I understand that cognitive data are the outcome variables, but it is not clear to me why so many variables have to be collected. Will all of them be used? How?

We appreciate the opportunity to clarify the reason for collecting a detailed cognitive battery. As discussed above in response to the previous reviewer, the cognitive battery is designed to be harmonised to other studies in the HCAP network. The goal is to provide comprehensive, harmonised data on cognitive health in longitudinal ageing studies. We have added an explanation of this in the Study Population section on Page 5 (see below).

<u>The NICOLA-HCAP protocol consists of a battery of neuropsychological tests and is designed to provide detailed data on ability across a range of cognitive domains, including memory, executive function, language and visuospatial ability. The protocol was originally developed and implemented within HRS and has since been implemented in several other national ageing cohorts (Lee et al., 2019; Mejia-Arango et al., 2020; Cadar et al., 2021; Bassil et al., 2022). The protocol is designed to be delivered in a harmonised way across studies to facilitate cross-national comparisons of dementia prevalence and risk factors</u>

The cognitive data collected will be used to provide algorithm-based research diagnoses of dementia or mild-cognitive impairment within the study sample. This involves utilising data

from all included tests to derive cognitive domain-level factor scores. We have added a summary of this approach on Page 16 in the section titled ‘Cognitive health outcome variables. The present study will take advantage of these diagnoses as outcomes (see below).

Following data collection and processing, domain-level factor scores derived from the scores on the cognitive battery will be used in conjunction with informant measures to develop algorithm-based research diagnoses of probable dementia or cognitive impairment. This approach has previously been used to estimate dementia prevalence in the HRS-HCAP sub-study (Manly et al., 2022). In a supplementary analysis, we will retrofit this predicted algorithmic score to the full Wave 1 and Wave 2 NICOLA longitudinal dataset.

Methods and analysis
Although the authors claim to follow the STROBE guidelines, I think there are some issues that could be clearer:

Study design
- In the "study design" section, the protocol talk about the objective, the cohort studies that will be used to conduct this protocol, how the first wave of this study will be recruited and the data that will be collected. I think that this section is not the place to give all these details. In my opinion they confuse the reader.

We have now removed the first sentence from the study design paragraph which highlighted the study aim as we recognised that it had already been stated within the introduction section (see page 5). In addition, we removed the sentence which detailed the data that will be collected as we agree this is not the place for this information (see page 5).

- I think this section should contain the type of design that is proposed for this study (what is it?) and depending on the design what groups there will be and if there will be follow-up. Having read the protocol several times, I am still not clear on these details.

We have now added an opening sentence to the Study design section and linked this to the timeline presented within the Supplementary files (see page 5).

- After the "study design" section, I would include a section (or several sections) on the target population, the accessible population and the study subjects: the sample, sampling technique, inclusion and exclusion criteria and recruitment. These sections should include the details found in the "study design" section.

- In the section on participant selection criteria, is it correct that there are no exclusion criteria?

We have restructured this section to provide the reader with details of the study population, sample size, participant recruitment and inclusion/exclusion criteria. Participant exclusion criteria has now been added (see page 6) and below:

Participant exclusion criteria:

1) Unable to complete the written NICOLA-HCAP assessment; and
2) Not fluent in English.

- Regarding the proposed sample size, no statistical criteria are used for the precision of the estimates or the power of the contrasts to be used in the analysis. This seems to me to be very important to address, firstly because it would clarify the exposure and outcome variables to be handled in the project, and secondly because it will allow the

feasibility of the project to be assessed. On the other hand, considering the number of exposure variables to be used and some types of analysis proposed on the multilevel SEM models, the size of 400 individuals seems to me to be a small sample size for the main objective of the protocol.

As detailed in the manuscript, we have outlined a number of analyses to address our objectives. The sample size described in the manuscript is for the mediation analysis. This is based on a sample size calculation to detect mediated effect as described by Fritz and MacKinnon. (see page 21 and 22).

- The flowchart in figure 1 describing the recruitment process has a double branch at the end which is not clear.

We have reviewed and amended Figure 1. The double branch has now been removed from the Figure.

Data collection and processing
- Regarding the groups of variables proposed to be used in the project, I think that an effort could be made to describe them in a clearer way. In addition, I have doubts that it is necessary to use so many variables for the objectives proposed in the project.

We have now reordered this section to make it clearer for the reader which groups of variables are being collected in this project. We have also made sure that it is clear how variables are derived (e.g., accelerometry versus data integration via HABITUS) (see pages 10-19).

- The role of the three groups of variables related to accelerometer, GIS and GPS data seems clear. These data are proposed to be integrated using the HABITUS tool, but it is not clear which variables are obtained from this tool to introduce into the analysis.

We have added further details about which variables will be derived following data integration via HABITUS and which will be derived by accelerometry alone (see pages 10-12).

- Regarding the molecular variables, it is not clear in my opinion what role they play in the protocol. For this type of variable, a multimodal analysis is proposed; is it possible to better clarify how this analysis will be carried out?

Please see our earlier response regarding multimodal analysis. Multimodal analysis is simply a methodological approach to comprehensively analyse different types of data together to provide a more holistic understanding of complex systems. In this way we can build a picture over multiple layers including different data modalities, identifying risk factors and patterns that would be missed when analysing each type of data (modality) separately. This approach also helps to identify biological interactions and regulatory dependencies.

- Regarding the variables in the "Cognitive Health Outcomes" group, a large number of instruments (scales) are described to measure the cognitive health of the study participants, as well as other instruments for the informants. Are so many variables necessary to assess cognitive health, would the MMSE not be sufficient, and how to justify the other variables? At the end of this section it is commented that "Following data collection and processing a research diagnosis of probable presence of absence of dementia and cognitive impairment will be ascertained using algorithms that will be developed in collaboration with colleagues from the Health and Retirement Study family of studies. In a supplementary analysis, we will retrofit this predicted algorithmic score to the full Wave 1 and Wave 2 NICOLA longitudinal dataset". This comment does not really explain what will be done with so many variables.

As discussed above, the process for deriving diagnoses using algorithms requires a broad range of cognitive measures to provide detailed information on a participant's cognitive ability. Data from these measures will be sued to derive factor scores based on cognitive domains – these factor scores are then fed into the algorithm to derive the diagnoses. This process has been used before in the HRS HCAP study – we have added a reference to this on page 16. Utilising a broad range of cognitive assessments will provide much more reliable estimates than using just the MMSE alone.

- I also think it would be more logical to have first described all the exposure variables (accelerometer, GIS and GPS), their integration, the molecular variables and finally the outcome variables on the cognitive health of the participants.

We have reordered this section (see pages 7-19).

- Finally, I think it would be clarifying to emphasise at the end of the section which are the outcome variables (dependent) and which are the exposure variables (independent) and possible confounders, mediators and/or moderators.

We have now gone through the data collection and processing section and emphasised at each section the variable type. In addition, we added a section providing details of confounders (see page 19).

Regarding the identification of the role of each variable, we feel this would not be best placed at the end of the section due to the role of each variable changing from one analysis to another – currently we have three different analyses planned. Therefore, we have taken the time to go through each of the analysis sections and for those that were missing detail/s relating to variable roles we have now provided additional detail (see pages 20-23). Furthermore, Figure 4 also provides an illustrative example.

Longitudinal follow up
In this section on follow-up, it is stated that a second wave of participants will be recruited using the NICOLA-HCAP study. It would be good to clarify:

- Is there any follow-up for the participants of the first wave?

This is a longitudinal study design. The same participants who completed wave 1 will take part in wave 2 with the exception of those who are now living in a nursing home, cannot provide written informed consent or those who have been deemed not to have the capacity to consent.

- Is this second wave a second group of participants, what is its purpose in the study, and does it have any relation to the first wave?

The participants being followed up are from the first wave. Participants (where possible) are being followed up longitudinally, so the purpose of the analysis is to investigate change over time and apply causal statistical approaches.

- It is said that this second wave will be recruited with the same inclusion/exclusion criteria. What are the exclusion criteria? No such criteria are stated.

This is a longitudinal study design so the same participants from wave 1 will be invited to participate in wave 2. As per the comment above we have now included participant exclusion criteria (see page 6). In addition, within the longitudinal follow up section we have also highlighted that for wave 2 if any participant from wave 1 is now living in a nursing home, cannot provide written informed consent or they have been deemed not to have the capacity to consent they will be excluded from wave 2.

Statistical analysis
Regarding this section:
- In the first approach the total effect of physical activity on cognitive function is analysed, where it is indicated that for cognitive function the MMSE scale will be used. Will the other variables in table 2 and table 3 also be used?

In the first instance the MMSE scale will be used, with the potential for additional analyses to be performed by the research team.

- Regarding the exposure factors, MVPA will be used. Are these variables the ones obtained from the integration using HABITUS? Which specific variables are the exposure variables?

As described within the data integration section (see page 11) HABITUS provides us with a method of integrating GIS, GPS and physical activity data to obtain variables relating to 'exposure through behaviour'. Following the reviewer comments, we have restructured this section to make it clearer and we have highlighted which variables are derived following data integration via HABITUS (see page 12). In addition, we have restructured the physical activity and sedentary behaviour section to ensure the reader is clear in regard to the variables which can be obtained via accelerometery (see page 10 and 11).

- It is stated that "Multivariable linear regression models with doubly robust estimators will be used to estimate the average causal effect", is this correct and is the dependent variable multivariate? On page 19, line 25 it says again "multivariate analysis of variance". What is the multivariate variable?

Through this study we are aiming to conduct a range of statistical analyses to test a range of research questions. Regarding page 19 line 25 (original manuscript) "multivariate analysis of variance" and the multivariate variable. For part 1 this is the different composition of behaviour (i.e., sedentary behaviour, light physical activity, and moderate-to-vigorous physical activity) and for part 2 it is the different composition of behaviour with the addition of geographical location (i.e., sedentary behaviour in green and blue space, sedentary behaviour everywhere else, light physical activity in green and blue space, light physical activity everywhere else, moderate-to-vigorous physical activity in green and blue space and moderate-to-vigorous physical activity everywhere else).

- On the other hand, isn't the model presented in figure 4 on the effect of physical activity on cognitive function a SEM model? And if so, aren't there variables in this figure that may act as confounders, mediators or moderators? Or perhaps this analysis is the one described in the next section "Mediation analysis"?

The model presented in Figure 4 is not included for Structural Equation Modeling (SEM) purposes. Instead, the purpose of this Directed Acyclic Graph (DAG) is to outline the general assumptions and to inform the estimation of the "total effect" of physical activity on cognitive function, as it is referred to in the text. It is just a preliminary illustration of the general categories of potential confounders rather than an attempt to identify specific individual variables. In our analysis, we will use the "backdoor criterion" to identify the causal effect, which is why the focus is on the confounder categories depicted in this DAG. To clarify, we added a legend to the figure to explicitly show the covariate roles.

Finally, no rough timeline for the protocol is included.

We have taken this comment on board in addition to a similar comment made by Reviewer 1. In light of these comments, we have added a Supplementary figure to the manuscript highlighting the study timeline. Please see Supplementary Figure 1.

VERSION 2 - REVIEW

**Reviewer**	**1**
**Name**	**Halpin, Amy**
**Affiliation**	**The University of Maine**
**Date**	**28-Oct-2024**
**COI**	

I appreciate the authors detailed and careful responses to my concerns. Most notably, I appreciate their explanation about the reasons behind choosing their neuropsychological battery, and the greater benefit of harmonizing data with other large aging studies. I continue to believe this is a sound and important study for the field of cognitive aging. I have no further concerns at this time.
